# β subunit affects Na^+^ and K^+^ affinities of Na^+^/K^+^-ATPase: Na^+^ and K^+^ affinities of a hybrid Na^+^/K^+^-ATPase composed of insect α and mammalian β subunits

**DOI:** 10.1016/j.bbrep.2022.101347

**Published:** 2022-09-14

**Authors:** Haruo Homareda, Kei Suga, Sachiko Yamamoto-Hijikata, Yoshinobu Eishi, Makoto Ushimaru, Yukichi Hara

**Affiliations:** a Department of Chemistry, Faculty of Medicine, Kyorin University, Shinkawa, Mitaka, Tokyo, 181-8611, Japan; b Department of Human Pathology, Graduate School and Faculty of Medicine, Tokyo Medical and Dental University, Yushima, Bunkyo-ku, Tokyo, 113-8519, Japan

**Keywords:** Silkworm, Rat, Hybrid Na^+^/K^+^-ATPase, Na^+^ affinity, K^+^ affinity, BM-N cell

## Abstract

The affinity for K^+^ of silkworm Na^+^/K^+^-ATPase, which is composed of α and β subunits, is remarkably lower than that of mammalian Na^+^/K^+^-ATPase, with a slightly higher affinity for Na^+^. Because the α subunit had more than 70% identity to the mammalian α subunit in the amino acid sequence, whereas the β subunit, a glycosylated protein, had less than 30% identity to the mammalian β subunit, it was suggested that the β subunit was involved in the affinities for Na^+^ and K^+^ of Na^+^/K^+^-ATPase. To confirm this hypothesis, we examined whether replacing the silkworm β subunit with the mammalian β subunit affected the affinities for Na^+^ and K^+^ of Na^+^/K^+^-ATPase. Cloned silkworm α and cloned rat β1 were co-expressed in BM-N cells, a cultured silkworm ovary-derived cell lacking endogenous Na^+^/K^+^-ATPase, to construct a hybrid Na^+^/K^+^-ATPase, in which the silkworm β subunit was replaced with the rat β1 subunit. The hybrid Na^+^/K^+^-ATPase increased the affinity for K^+^ by 4.1-fold and for Na^+^ by 0.65-fold compared to the wild-type one. Deglycosylation of the silkworm β subunit did not affect the K^+^ affinity. These results support the involvement of the β subunit in the Na^+^ and K^+^ affinities of Na^+^/K^+^-ATPase.

## Highlights

• Silkworm Na^+^/K^+^-ATPase has much lower affinity for K^+^ than mammalian Na^+^/K^+^-ATPase with a slightly higher affinity for Na^+^.
• Silkworm Na^+^/K^+^-ATPase β subunit has less than 30% identity to the mammalian β subunit in the amino acid sequence.
• Replacement of the silkworm β with the rat β increased K^+^ affinity and decreased Na^+^ affinity of Na^+^/K^+^-ATPase.
• β subunit is involved in Na^+^ and K^+^ affinities of Na^+^/K^+^-ATPase.

## Abbreviations

Sα: silkworm Na^+^/K^+^-ATPase α subunit expressed in BM-N cells
Sβ: silkworm Na^+^/K^+^-ATPase β subunit expressed in BM-N cells
Rβ: rat Na^+^/K^+^-ATPase β1 subunit expressed in BM-N cells
SαSβ: Na^+^/K^+^-ATPase composed of Sα and Sβ
Sα/Rβ: Na^+^/K^+^-ATPase composed of Sα and Rβ
PNGase F: glycopeptidase F
*K*_0.5_ value for K^+^: concentration of K^+^ giving the half-maximal enzyme activity
*K*_0.5_ value for Na^+^: concentration of Na^+^ giving the half-maximal enzyme activity

## 1 Introduction

Na^+^/K^+^-ATPase (EC.3.6.3.9) is a membrane-bound protein complex actively transporting Na^+^ from the inside to the outside of the cell and inversely transporting K^+^ in most animal cells [1,2]. Defects in Na^+^/K^+^-ATPase cause serious disorders in invertebrates as well as vertebrates [[2], [3], [4], [5], [6]]. This protein complex is composed of two major subunits, α and β, and in some tissues, γ (FXYD), a small membrane protein [7]. The central function of the α subunit is transport of Na^+^ and K^+^ coupled with hydrolysis of ATP. The β subunit, a glycosylated protein, has been thought to support the α subunit in plasma membranes [1,2]. However, some investigators have reported more active involvements of the β subunit in Na^+^/K^+^-ATPase activity and ion transport. Kawamura et al. [8] indicated that a reduction of disulfide bonds in the β subunit caused loss of ATPase activity. Lutsenko and Kaplan [9] reported that a reduction of disulfide bonds in the β subunit caused the loss of K^+^ occlusion. Geering et al. [10] reported that the affinities for Na^+^ and K^+^ of Na^+^/K^+^-ATPase varied with the various combinations of α (α1–α4) and β isoforms (β1–β3).

The Na^+^: K^+^ ratio in the hemolymph of Lepidopterous insects is 1:5–10 [11], which is the directly opposite that (10:1) in mammalian blood. The Na^+^ and K^+^ concentrations in the hemolymph of silkworm, *Bombyx mori*, a Lepidopterous insect, are reported to be 14.6 mM Na^+^ and 46.1 mM K^+^ [12]. The Na^+^: K^+^ ratio, therefore, is 1:3. We found that the silkworm has abundant Na^+^/K^+^-ATPase in its central nervous system but not in other tissues, and the affinity for K^+^ of silkworm Na^+^/K^+^-ATPase was much lower than that of mammalian Na^+^/K^+^-ATPase with a slightly higher affinity for Na^+^ [13]. The concentration of Na^+^ giving the half-maximal enzyme activity (*K*_0.5_ value for Na^+^) and the concentration of K^+^ giving the half-maximal enzyme activity (*K*_0.5_ value for K^+^) of mammalian Na^+^/K^+^-ATPase were 8 and 1.5 mM in the presence of 10 mM KCl and 100 mM NaCl, respectively. On the other hand, the *K*_0.5_ value for Na^+^ and the *K*_0.5_ value for K^+^ of silkworm Na^+^/K^+^-ATPase were 5 and 6 mM in the presence of 10 mM KCl and 100 mM NaCl, respectively. The K^+^ affinity of silkworm Na^+^/K^+^-ATPase was lower than that of mammalian one at 10 mM NaCl [13]. In addition, the *K*_0.5_ values for K^+^ of mammalian and silkworm K^+^-dependent pNPPase activity were 0.6 and 6.0 mM, respectively [13].

We cloned cDNAs of Na/K-ATPase α and β subunits in the silkworm nervous system, and analyzed the deduced amino acid sequences. The α subunit had more than 70% identity with mammalian α1–α4 isoforms, while the β subunit had less than 30% identity with mammalian β1–β3 isoforms and the apparent molecular mass of silkworm β subunit was lower than that of mammalian β subunit [15]. These results suggest that the β subunit is involved in the affinities for Na^+^ and K^+^ of Na^+^/K^+^-ATPase.

To verify this hypothesis, a hybrid Na^+^/K^+^-ATPase, which was composed of a silkworm α subunit and a rat β1 subunit, and a wild-type Na^+^/K^+^-ATPase, which was composed of silkworm α and β subunits, were expressed using silkworm α cDNA, silkworm β cDNA, and rat β1cDNA in BM-N cells, a cultured silkworm ovary-derived cell lacking endogenous Na^+^/K^+^-ATPase [15]. The hybrid Na^+^/K^+^-ATPase increased the affinity for K^+^ but decreased the affinity for Na^+^ compared to a wild-type one.

## 2 Materials and Methods

### 2.1 Materials

Silkworm larvae in the fifth instar feeding stage were obtained from the Institute of Genetic Resources, Faculty of Agriculture, Kyushu University (Fukuoka, Japan) and Ehime Sanshu Co. Ltd. (Yawatahama, Japan). Female Wistar rats were obtained from Japan SLC, Inc. (Hamamatsu, Japan). All of the experimental procedures using animals were approved by the Experimental Animal Ethics Committee of Kyorin University, and performed in accordance with the guidelines for handling laboratory animals. BM-N cells were purchased from Riken Bioresource Center (Tsukuba, Japan). PVDF membranes were purchased from Bio-Rad Laboratories, Inc. (Hercules, CA, USA). ECL Prime Western Blotting Reagents were purchased from GE Healthcare (Little Chalfont, Buckinghamshire, England). Cloned glycopeptidase F (PNGase F) was purchased from Takara Bio Inc. (Tokyo, Japan). A non-interacting protein assay kit was purchased from Calbiochem (San Diego, USA). A Wide-View Prestained Protein Size Marker, protease inhibitor mixture and other reagents were purchased from FUJIFILM Wako Pure Chemicals Corporation (Osaka, Japan).

### 2.2 Methods

#### 2.2.1 Construction and expression of plasmids

Silkworm α and β cDNAs were cloned as described in a previous paper [15]. Rat β1 cDNA was cloned in our laboratory. Flag-tagged silkworm α subunit cDNA and HA-tagged silkworm β cDNA were inserted into BM-N cells to express a wild-type (SαSβ) of silkworm Na^+^/K^+^-ATPase, which is composed of the α subunit (Sα) and β subunit (Sβ) of silkworm Na^+^/K^+^-ATPase. Flag-tagged silkworm α subunit cDNA and HA-tagged rat β1 cDNA were inserted into BM-N cells to express a hybrid-type (Sα/Rβ) of Na^+^/K^+^-ATPase, which is composed of Sα and the β1 subunit (Rβ) of rat Na^+^/K^+^-ATPase, and the transfected cells were cultured as described in a previous paper [15].

#### 2.2.2 Preparation of microsomes

The method of preparing microsomes of the silkworm nerve tissue and rat kidney was slightly modified from the previous method [13]. Briefly, the nerve tissue was isolated from the abdominal side under a stereoscope and suspended in ice-cold preparation solution (5 mM DTT, 5 mM EDTA, 250 mM sucrose, protease inhibitors and 50 mM imidazole-HCl, pH 7.6), minced with scissors, homogenized with a glass homogenizer, and centrifuged at 2,500 g for 20 min at 5 °C. Supernatants were centrifuged at 100,000 g for 20 min at 5 °C. Precipitants were suspended at 1 mM EDTA and stored in liquid nitrogen.

Microsomes of BM-N cells expressing SαSβ and Sα/Rβ were prepared as described in a previous paper [15].

#### 2.2.3 Antiserum and antibody

Anti-dog Na^+^/K^+^-ATPase α subunit antiserum and anti-dog Na^+^/K^+^-ATPase β subunit antiserum were prepared using purified canine kidney Na^+^/K^+^-ATPase α and β subunits as described in a previous paper [16]. Anti-silkworm Na^+^/K^+^-ATPase β subunit antibody was prepared by Sigma-Aldrich Japan as described in a previous paper [15].

The secondary antibody, anti-rabbit IgG, horseradish peroxidase linked whole antibody (from donkey, NA934), was purchased from GH Healthcare.

#### 2.2.4 SDS-PAGE and immunoblotting

Microsomes (4–50 μg) of silkworm nerve, SαSβ, rat kidney, and Sα/Rβ were applied to SDS-PAGE with 9% or 10.5% acrylamide and then blotted onto a PVDF membrane as described previously [[13], [14], [15]]. The blotted membrane was incubated with primary antibodies, a 1:1000 dilution of anti-dog Na^+^/K^+^-ATPase α subunit antiserum, a 1:500 dilution of anti-dog Na^+^/K^+^-ATPase β subunit antiserum, or a 1:1000 dilution of anti-silkworm Na^+^/K^+^-ATPase β subunit antibody for 1 h at room temperature, washed, and then incubated with the secondary antibody, a 1:10,000 dilution of anti-rabbit IgG, horseradish peroxidase linked whole antibody for 20 min at room temperature, chemiluminesced with ECL Prime Western Blotting Reagents, and visualized using a CCD camera (Image Quant LAS-4000, GE Healthcare) [15]. The marker proteins were electrophoresed with the target proteins. The lane of marker proteins was separated from the lane of the target proteins, stained with Coomassie brilliant blue, and photographed with a CCD camera. These two images were composed into a figure with a computer software.

#### 2.2.5 Treatment with PNGase F

To release oligosaccharides from the β subunit, microsomes (4–50 μg) of silkworm nerve, rat kidney, SαSβ, and Sα/Rβ were treated with 0.25 mU PNGase F at 37 °C for 3 h in 20–28 μl of the denaturing buffer (0.14% SDS, 1% Triton X-100, 10 mM EDTA, 2% mercaptoethanol, and 50 mM Tris/HCl, pH 8.6). The reaction was stopped by freezing the reaction mixture. For measurement of Na^+^/K^+^-ATPase activity, microsomes (20 μg) of silkworm nerve were treated with 0.4 mU PNGase F at 30 °C for 3 h in 20 μl of the native buffer (50 mM Tris/HCl, pH 8.6). The treated microsomes (1 μg) were directly added to the ATPase reaction mixture (60 μl).

#### 2.2.6 Assay of Na^+^/K^+^-ATPase activity

The standard reaction mixture for silkworm Na^+^/K^+^-ATPase activity is composed of 10 mM NaCl, 30 mM KCl, 4 mM MgCl_2_, 1 mM EDTA, 2 mM ATP, and 50 mM imidazole-HCl (pH 7.2 at 37 °C) [13,15], and microsomes (6 μg) of SαSβ or Sα/Rβ, or microsomes (1 μg) of silkworm nerve tissue. For measurement of the Na^+^ affinity, the mixture contained 0–100 mM NaCl and 30 mM KCl. The mixture for measurement of the K^+^ affinity contained 10 mM NaCl and 0–100 mM KCl. The ATPase reaction was started by the addition of ATP. The reaction mixture was incubated for 60 min at 37 °C, and stopped by addition of H_2_SO_4_-molybdate solution according to Fiske-Subbarow Method [17]. Liberated and colored inorganic phosphate was assayed by a microplate reader (iMark, BioRad Laboratories, Inc.). The difference between the activities in the presence and absence of both NaCl and KCl was defined as Na^+^/K^+^-ATPase activity.

#### 2.2.7 Protein concentration assay

Protein concentrations of microsomes were determined by a non-interfering assay kit, using bovine serum albumin as a standard.

## 3 Results and discussion

### 3.1 Characterization of β subunits expressed in BM-N cells

BM-N cells do not have Na^+^/K^+^-ATPase subunits and ouabain-sensitive ATPase activity as shown in a previous paper [Figs. 5 and 7 of Ref. 15]. Therefore, we used this cultured cell as a host cell to express SαSβ and Sα/Rβ.

**Fig. 1:**
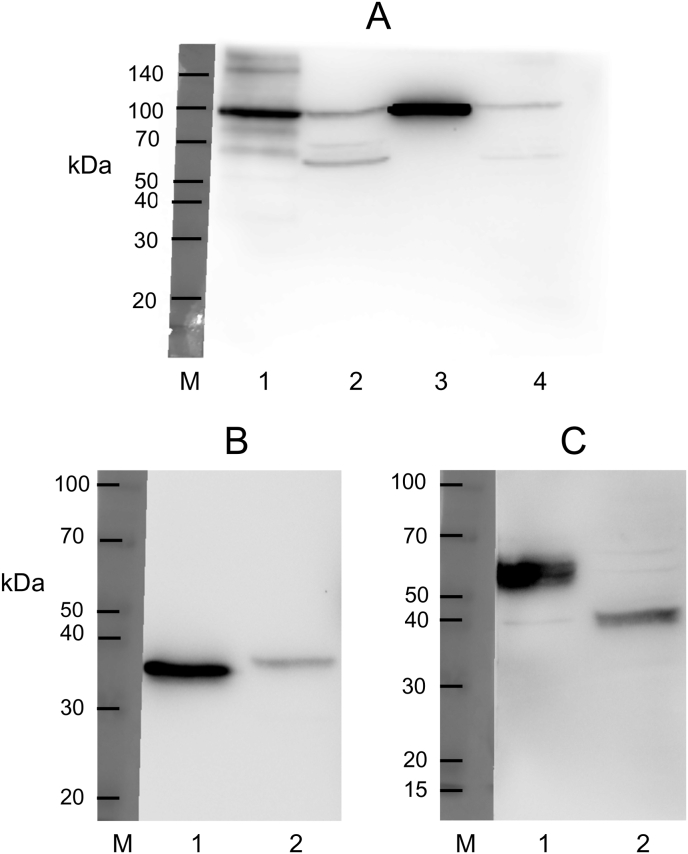
(A) Expression of Na^+^/K^+^-ATPase α subunit in silkworm nerve, SαSβ, rat kidney, and Sα/Rβ microsomes. Silkworm nerve (18 μg) (lane 1), SαSβ (50 μg) (lane 2), rat kidney (4 μg) (lane 3), and Sα/Rβ microsomes (50 μg) (lane 4) were applied to SDS-PAGE with 9% acrylamide, blotted onto a PVDF membrane, and incubated with a 1:1000-dilution of anti-dog Na^+^/K^+^-ATPase α subunit antiserum for 1 h at room temperature. (B) Expression of Na^+^/K^+^-ATPase β subunit in silkworm nerve and SαSβ microsomes. Silkworm nerve (25 μg) (lane 1) and SαSβ (35 μg) (lane 2) were applied to SDS-PAGE with 10.5% acrylamide, blotted onto a PVDF membrane, and incubated with a 1:1000-dilution of anti-silkworm Na^+^/K^+^-ATPase β subunit antibody. (C) Expression of Na^+^/K^+^-ATPase β subunit in rat kidney and Sα/Rβ microsomes. Rat kidney (13 μg) (lane 1) and Sα/Rβ (30 μg) (lane 2) were applied to SDS-PAGE with 10.5% acrylamide, blotted onto a PVDF membrane, and incubated with a 1:500-dilution of anti-dog Na^+^/K^+^-ATPase β subunit antiserum for 1 h at room temperature. The subsequent procedure was described in Materials and Methods. Lane M represents the molecular mass marker.

**Fig. 2:**
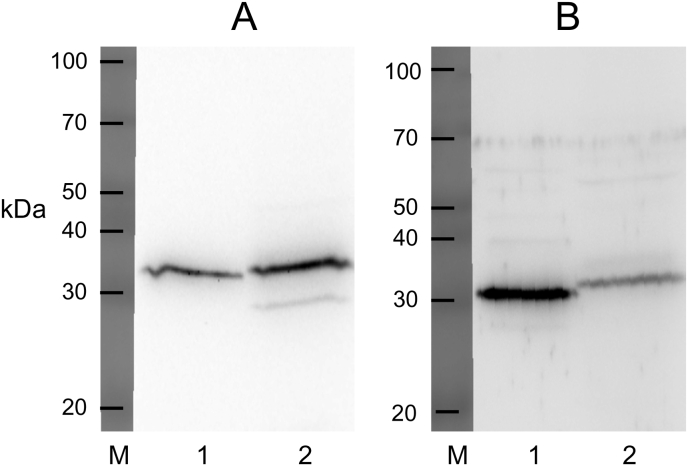
(A) PNGase F-treated Na^+^/K^+^-ATPase β subunits in silkworm nerve and SαSβ microsomes. Silkworm nerve (4 μg) (lane 1) and SαSβ (50 μg) (lane 2), which were treated with PNGase F in the denaturing buffer as described in Materials and Methods, were applied to SDS-PAGE with 10.5% acrylamide, and blotted onto a PVDF membrane. The blotted membranes were incubated with a 1:1000-dilution of anti-silkworm Na^+^/K^+^-ATPase β subunit antibody for 1 h at room temperature. (B) PNGase F-treated Na^+^/K^+^-ATPase β subunits in rat kidney and Sα/Rβ microsomes. Rat kidney (6 μg) (lane 1) and Sα/Rβ (40 μg) (lane 2), which were treated with PNGase F as described in Materials and Methods, were applied to SDS-PAGE with 10.5% acrylamide, and blotted onto a PVDF membrane. The blotted membranes were incubated with a 1:500-dilution of anti-dog Na^+^/K^+^-ATPase β subunit antiserum for 1 h at room temperature. The subsequent procedures were described in Materials and Methods. Lane M represents the molecular mass marker.

The molecular masses of the Sα subunits in SαSβ and Sα/Rβ expressed in BM-N cells were 100 kDa, although some non-specific bands smaller than 70 kDa were observed (Fig. 1A, lanes 2 and 4). The molecular mass of the Sβ subunit in SαSβ, 37 kDa, was almost the same as that of the silkworm β, 35 kDa (Fig. 1B), taking the HA-tag (eleven amino acid peptide) of Sβ into consideration. On the other hand, the molecular mass of the Rβ subunit in Sα/Rβ, 39 kDa, was much smaller than that of the rat kidney β1, 57 kDa (Fig. 1C). After PNGase F treatment at 37 °C for 3 h in the denaturing buffer (Supplementary Fig. 1), molecular masses of their β subunits decreased to 31–33 kDa (Fig. 2A and B), which are the molecular mass of the deglycosylated β subunit [18,19].

Fig. 2 suggests that the oligosaccharides bound with silkworm β subunits were the N-linked glycosylation type, like those binding with rat β1 subunit, because PNGase F specifically cleaved the GlcNAc-Asn bond.

It is known that the molecular mass of a glycoprotein on SDS-PAGE apparently varies with the number and/or structure of oligosaccharides [18,19]. The structure of oligosaccharides of Lepidopterous insect cells is different from that of mammalian cells due to the difference in glycosyltransferase [[20], [21], [22]]. The number of oligosaccharides of the silkworm β subunit was smaller than that of the rat β1 subunit [1,15]. Considering these data together, Fig. 1C suggests that the oligosaccharides of Rβ were different from those of the rat β1 in structure and/or number.

We examined whether replacement of the silkworm β with the Rβ affected the Na^+^ and K^+^ affinities of Na^+^/K^+^-ATPase.

### 3.2 Na^+^ and K^+^ affinities of a hybrid Na^+^/K^+^-ATPase

**Fig. 3:**
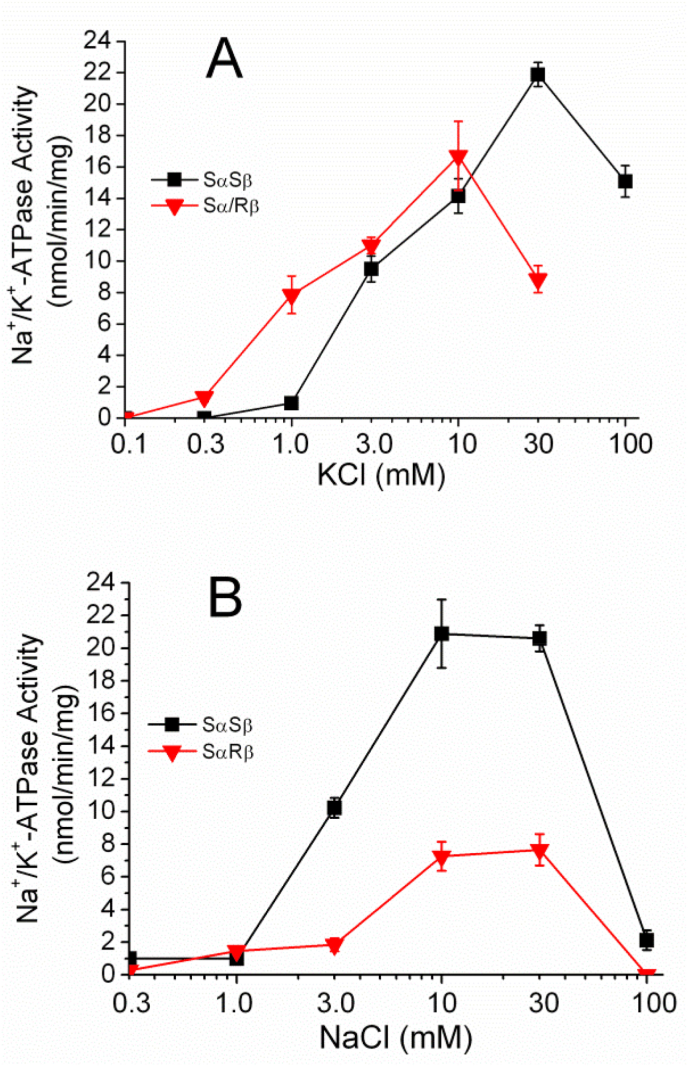
Affinities for K^+^ and Na^+^ of SαSβ and Sα/Rβ Na^+^/K^+^-ATPase. The reaction mixture was composed of (A) 10 mM NaCl, 0–100 mM KCl, (B) 0–100 mM NaCl, 30 mM KCl, and 4 mM MgCl_2_, 1 mM EDTA, 2 mM ATP, 50 mM imidazole/HCl (pH 7.2 at 37 °C), and 6 μg of SαSβ (■) and Sα/Rβ microsomes (▼) in 60 μl. The following procedure was described in Materials and Methods. Data are represented as mean ± S.E. (n = 4).

The *K*_0.5_ values for K^+^ of SαSβ and Sα/Rβ Na^+^/K^+^-ATPase were 4.5 mM and 1.1 mM, respectively (Fig. 3A), This result indicated that the replacement of silkworm β with the Rβ increased the affinity for K^+^ of Na^+^/K^+^-ATPase by 4.1-fold. On the other hand, the *K*_0.5_ values for Na^+^ of SαSβ and Sα/Rβ Na^+^/K^+^-ATPase were 3.0 mM and 4.6 mM, respectively (Fig. 3B) showing that the replacement of silkworm β with the Rβ increased the affinity for Na^+^ by 0.65-fold.

**Fig. 4:**
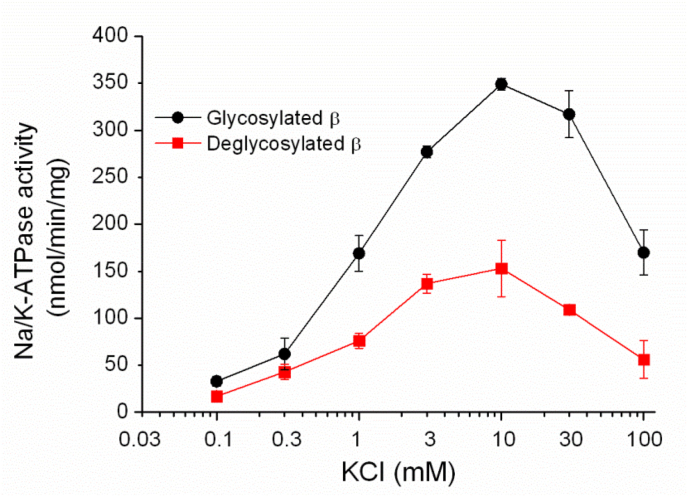
Affinity for K^+^ of silkworm Na^+^/K^+^-ATPase with glycosylated or deglycosylated β subunit. The reaction mixture was composed of 10 mM NaCl, 0–100 mM KCl, 4 mM MgCl_2_, 1 mM EDTA, 2 mM ATP, 50 mM/HCl (pH 7.2 at 37 °C), and 1 μg of silkworm Na^+^/K^+^-ATPase microsomes (●) or PNGase F treated-silkworm Na^+^/K^+^-ATPase microsomes (■), which were treated in the native buffer as described in Materials and Methods, in 60 μl. The following procedure was described in Materials and Methods. Data are represented as mean ± S.E. (n = 3).

Whether the oligosaccharides bound with the β subunit affected the affinity of K^+^ for silkworm Na^+^/K^+^-ATPase was examined (Fig. 4). The silkworm Na^+^/K^+^-ATPase was treated with PNGase F at 30 °C for 3 h in the native buffer (Supplementary Fig. 2). The *K*_0.5_ value for K^+^ of silkworm Na^+^/K^+^-ATPase was 1 mM, regardless of whether the β subunit was deglycosylated or not. This result indicated that glycosylation of the β subunit did not affect the K^+^ affinity of Na^+^/K^+^-ATPase activity.

Several studies have reported that various combinations of α and β isoforms have different effects on the affinities for K^+^ and Na^+^ of Na^+^/K^+^-ATPase. Habeck et al. [23] and Crambert et al. [24] showed that the replacement of β2 with β1 increased the K^+^ affinity of human Na^+^/K^+^-ATPase by 1.1–2.7-fold and increased the Na^+^ affinity by 0.5–0.7-fold. Jaisser et al. [25] indicated that the replacement of β1 with β3 increased the external K^+^ affinity of amphibian Na^+^/K^+^-ATPase, which is composed of *B. marinus* α/*X. laevis* β, by 1.9-fold. Shinoda et al. [26] suggested the necessity of the β subunit for K^+^ binding from analysis of the crystal structure of shark rectal gland Na^+^/K^+^-ATPase. Hilbers et al. [3] suggested that the relative orientation of the β subunit in the transmembrane domain of the α-β complex modulated ion binding at the α subunit. These reports also indicate the involvement of the β subunit in the Na^+^ and K^+^ affinities of Na^+^/K^+^-ATPase.

In this study, we demonstrated that the Rβ subunit can replace silkworm β subunit, affecting the Na^+^ and K^+^ affinities, although silkworm β subunit had less than 30% identity with mammalian β subunit in amino acid sequence, and the oligosaccharides of β subunit did not affect the K^+^ affinity. The mechanism of how the β subunit is involved in the affinities for Na^+^ and K^+^ is unknown. Analysis of the crystal structure of silkworm Na^+^/K^+^-ATPase will be necessary to elucidate this mechanism.

In Lepidopterous insects with a hemolymph with low Na^+^ and high K^+^ concentrations, Na^+^ can be substituted by K^+^. For example, the K^+^ pump (V-type ATPase), which does not require Na^+^, is present in Lepidopterous insects [27]. Unlike mammals, K^+^ pumped out of the cells by the pump is co-transported with amino acids into the midgut. Although the silkworm has such a system, Na^+^/K^+^-ATPase is essentially present in its nerve tissue and probably maintains the neuron resting potential and the ionic balance, adapting to the surrounding of low Na^+^ and high K^+^ concentrations [28]. These facts would emphasize how important Na^+^/K^+^-ATPase is to insects. In addition, the ability to exchange the β subunit between different species may indicate the physiological importance of Na^+^/K^+^-ATPase across species.

## Funding

This research did not receive any specific grant from funding agencies in the public, commercial, or not-for-profit sectors.

## Declaration of competing interest

The authors declare that they have no known competing financial interests or personal relationships that could have appeared to influence the work reported in this paper.
