# Supplementary material for: β subunit affects Na+ and K+ affinities of Na+/K+-ATPase: Na+ and K+ affinities of a hybrid Na+/K+-ATPase composed of insect α and mammalian β subunits
Source: Biochem Biophys Rep. 2022 Sep 14;32:101347. doi: 10.1016/j.bbrep.2022.101347 (PMC9483571; doi:10.1016/j.bbrep.2022.101347)
Supplement: Multimedia component 1 [file mmc1.pptx]

## Slide 1
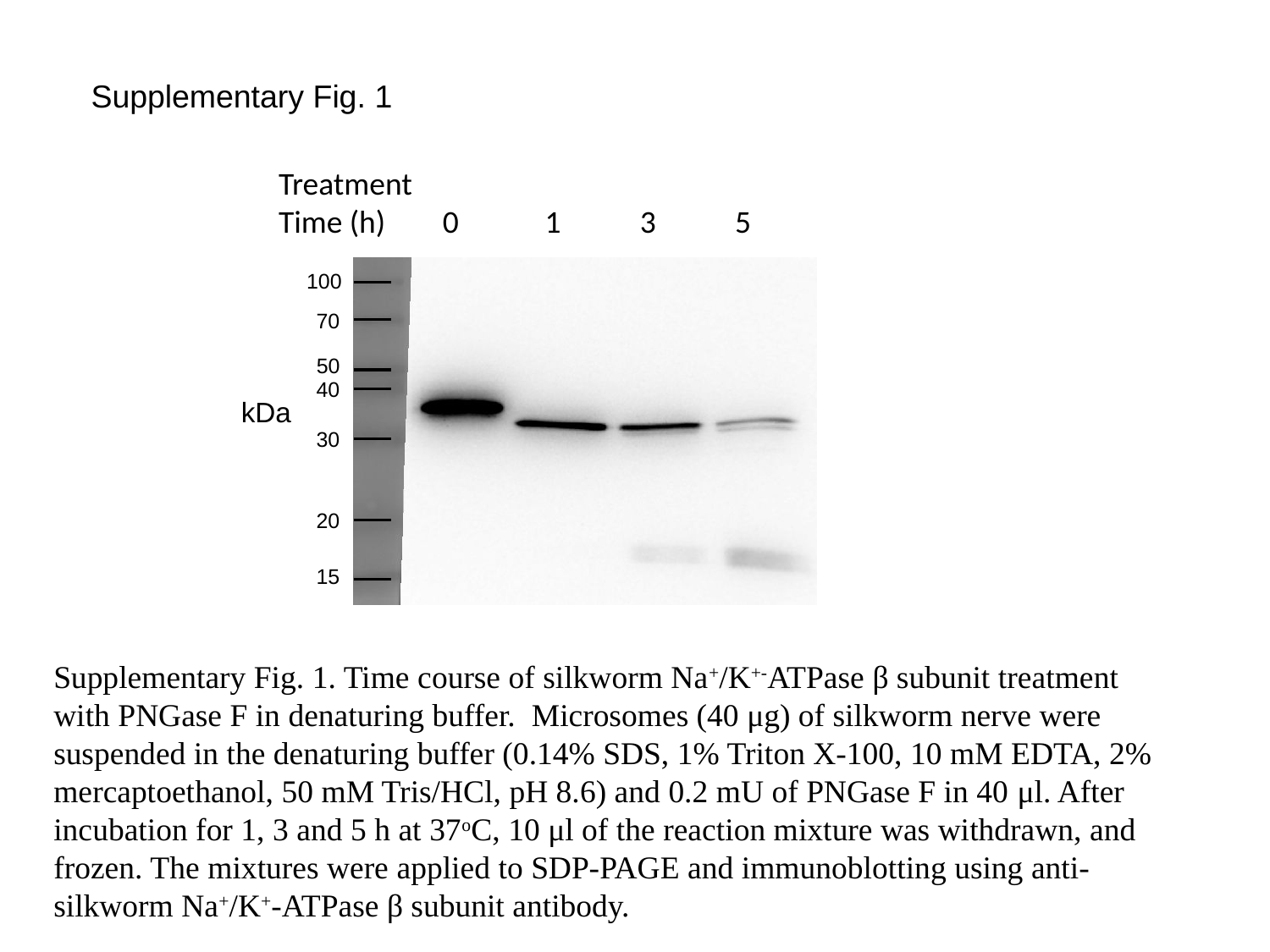

Supplementary Fig. 1
Treatment
Time (h) 0 1 3 5
100
70
50
40
kDa
30
20
15
Supplementary Fig. 1. Time course of silkworm Na+/K+-ATPase β subunit treatment with PNGase F in denaturing buffer. Microsomes (40 μg) of silkworm nerve were suspended in the denaturing buffer (0.14% SDS, 1% Triton X-100, 10 mM EDTA, 2% mercaptoethanol, 50 mM Tris/HCl, pH 8.6) and 0.2 mU of PNGase F in 40 μl. After incubation for 1, 3 and 5 h at 37oC, 10 μl of the reaction mixture was withdrawn, and frozen. The mixtures were applied to SDP-PAGE and immunoblotting using anti-silkworm Na+/K+-ATPase β subunit antibody.

## Slide 2
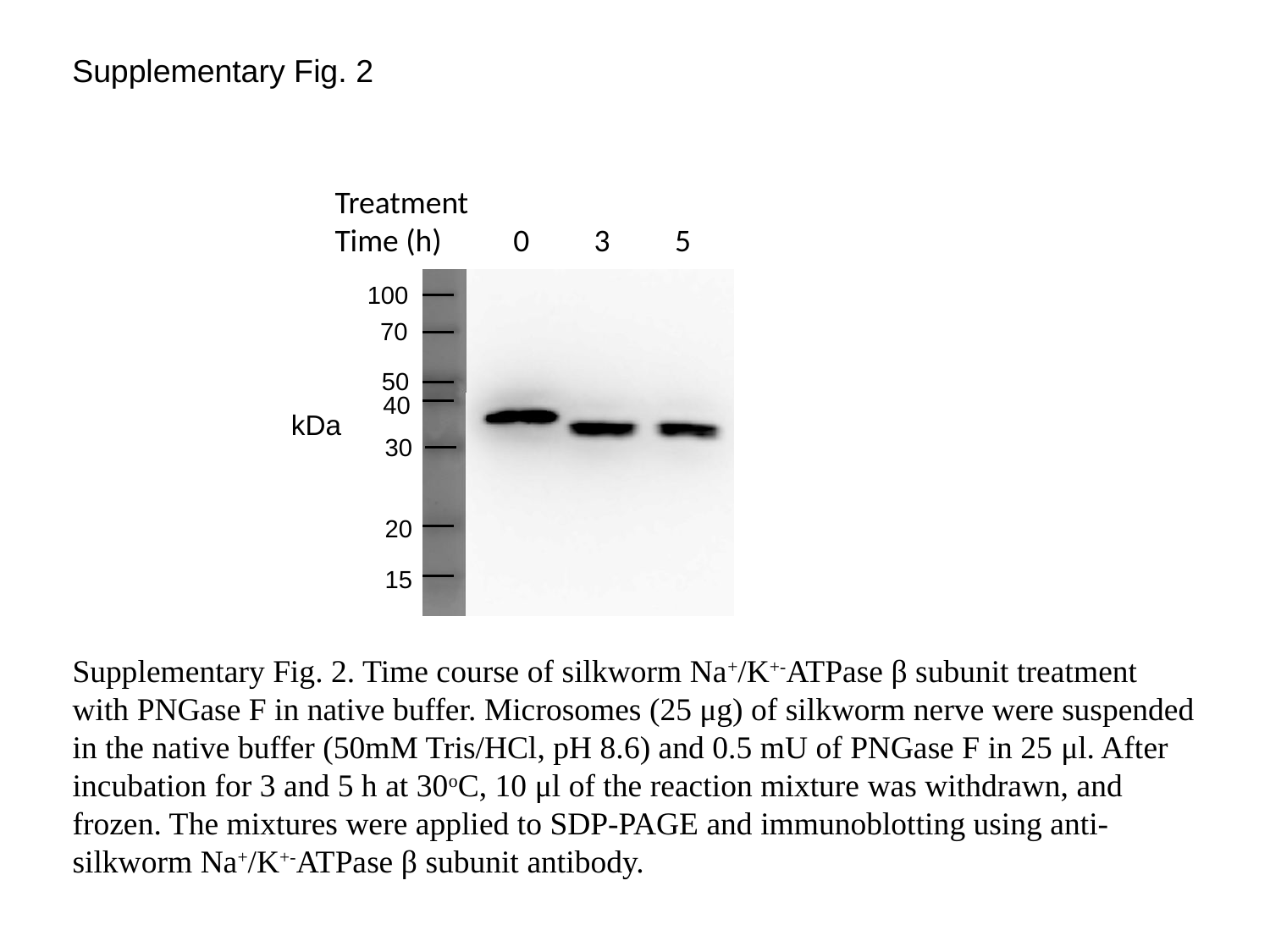

Supplementary Fig. 2
Treatment
Time (h) 0 3 5
100
70
50
40
kDa
30
20
15
Supplementary Fig. 2. Time course of silkworm Na+/K+-ATPase β subunit treatment with PNGase F in native buffer. Microsomes (25 μg) of silkworm nerve were suspended in the native buffer (50mM Tris/HCl, pH 8.6) and 0.5 mU of PNGase F in 25 μl. After incubation for 3 and 5 h at 30oC, 10 μl of the reaction mixture was withdrawn, and frozen. The mixtures were applied to SDP-PAGE and immunoblotting using anti-silkworm Na+/K+-ATPase β subunit antibody.
